# Immunogenicity of subunit vaccine of E2 protein against atypical porcine pestivirus in pigs

**DOI:** 10.3389/fcimb.2025.1740259

**Published:** 2026-01-06

**Authors:** Yufeng Huang, Lili Guo, Ling Chen, Lingling Yin, Peixun Yuan, Yongda Zhao

**Affiliations:** 1 College of Veterinary Medicine, Qingdao Agricultural University, Qingdao, China; 2 Qingdao Bolin Biotechnology Co., Ltd, Qingdao, China; 3 Shandong Center for Quality Control of Feed and Veterinary Drugs, Jinan, China; 4 Shandong Provincial Livestock Product Quality and Safety Center, Jinan, China

**Keywords:** antibody, APPV, E2, Escherichia coli expressing, vaccine

## Abstract

**Introduction:**

An atypical porcine pestivirus (APPV) causes congenital tremor (CT) type A-II in piglets and has caused considerable financial losses to the pig industry worldwide. This study aimed to develop an effective vaccine candidate against APPV.

**Methods:**

We constructed a prokaryotic expression system for the APPV E2 protein in Escherichia coli. The immunogenicity of the APPV E2 subunit vaccine, formulated with three different adjuvants (ISA 201VG, IMS 1313VG, and Gel 02), was evaluated in piglets. Assessment was based on E2-specific antibody levels measured by an in-house enzyme-linked immunosorbent assay (ELISA) and protection against an APPV challenge. Successful expression of the E2 protein was confirmed by western blot analysis.

**Results:**

The E2 protein was successfully expressed, and an E2-ELISA detection method was established. Vaccinated piglets mounted virus-specific IgG responses. The E2 protein emulsified with ISA 201VG and Gel 02 adjuvants induced significantly higher antibody levels than the formulation with IMS 1313VG adjuvant. The vaccine with ISA 201VG adjuvant yielded the highest antibody levels and provided protection against APPV infection. Histopathological examination confirmed normal organ morphology in vaccinated pigs, whereas the control group displayed significant pathological alterations.

**Discussion:**

The results demonstrate that the Escherichia coli-expressed E2 protein elicits an immune response in pigs. The E2 subunit vaccine is a potential candidate for preventing APPV infection, and ISA 201VG and Gel 02 adjuvants are suitable candidate adjuvants for vaccine preparation. This study provides a foundation for further APPV vaccine research.

## 1 Introduction

APPV is the causative agent of type A-I I congenital tremor (CT) in neonatal piglets and is characterized by generalized body shaking with variable degrees of hypomyelination in the brain and spinal cord (Hause et al., 2015; Gatto et al., 2019; Pan et al., 2020). Atypical porcine pestiviruses (APPV) were also detected in many countries, including the United States (Hause et al., 2015), Germany (Michelitsch et al., 2019), Poland (Augustyniak et al., 2025), Austria (Schwarz et al., 2017), and Spain (Muñoz-González et al., 2017). Moreover, APPV has been discovered in swine herds from China (Yuan et al., 2017), suggesting a wide distribution in the country. Since the disease mainly affects newborn piglets, vaccines have become the main effective means of combating the virus.

APPV is a pestivirus with an approximately 11 kB single-stranded positive-sense RNA genome. Its structural proteins include three envelope glycoproteins (Erns, E1, and E2), among which the principal E2 component, at 241 amino acids in length, is notably shorter than the E2 proteins of other pestiviruses (Hause et al., 2015). CSFV and APPV also belonged to the genus Pestivirus of the family Flaviviridae (Smith et al., 2017). E2 was the main protective antigen of the classical swine fever virus (CSFV) and was generally considered the main factor in developing the vaccines (Gao et al., 2020). Smit and Bouma founded that the gene-engineered subunit vaccine against the CSFV E2 protein has good stability and immune effect, and its antibody level is higher than that of the attenuated vaccine, which can effectively prevent and control CSFV infection (Bouma et al., 1999; de Smit et al., 2001). In view of these, E2 has also become the focus of APPV research. In 2018, Zhang et al. successfully developed an effective subunit vaccine against APPV based on the E2 protein expressed by the baculovirus expression system and induced a better Th2-type immune response in mice.

Montanidetm ISA 201 VG, Montanide™ IMS 1313, and Montanide Gel 02 adjuvant were produced by SEPPIC. They are all currently used for various vaccines, particularly for cattle, pigs, and small ruminants, and to induce enhanced immune responses and protective efficacy (de Smit et al., 2001; Dar et al., 2013; Osman et al., 2020). However, they each have their characteristics and advantages. Montanidetm ISA 201 VG is an adjuvant based on mineral oil for the preparation of oil-in-water emulsions. It contains special light mineral oils and highly refined emulsifiers of plant-derived mannitol and oleic acid. Montanide™ IMS 1313, a nanoparticle adjuvant, is an easily diluted adjuvant containing microemulsions and generally recognized as safe immune stimulation complexes. Montanide Gel 02 was a newly developed polymer adjuvant, which was aqueous dispersions of high molecular weight polyacrylic acid (Dar et al., 2013; Osman et al., 2020), contained nothing of animal origin, was a water-soluble adjuvant with good compatibility. Zhang et al. (2018) reported that the E2 protein emulsified with ISA 201VG adjuvant induced significantly higher levels of APPV-specific antibodies and elicited stronger lymphocyte proliferative responses and higher interleukin-10 secretion than those of the E2 protein emulsified with IMS 1313VG adjuvant.

In this study, a prokaryotic expression system for the APPV E2 protein was constructed in *Escherichia coli*. The system was used to evaluate the immune responses induced by the E2 protein in pigs and to assess the effects of different adjuvants (ISA 201VG, GEL02, and IMA 1313) in an APPV E2-subunit vaccine. Immune responses were measured by E2-specific ELISA antibody levels, and protection was evaluated against an APPV challenge. Thus, this study provides a theoretical basis for selecting candidate APPV vaccines.

## 2 Materials and methods

### 2.1 Genetic evolution analysis

We amplified the E2 gene by PCR using the primers F: AGCTGCATCAAACGTCAGGATTA and R: GTGACGGTACATGGTCGCCG. Sequences of APPV from different countries were processed by the Clustal W method, and phylogenetic trees were constructed by the neighbor-joining method using MEGA 7 software. Bootstrap values were indicated for each node from 1000 replicates. Additionally, 48 reference strains were chosen from Genbank for inclusion in the phylogenetic analysis ( **Supplementary Table S1**).

### 2.2 The construction of the plasmid pET28a-APPV E2

According to the E2 sequences of APPV obtained from disease pigs infected APPV (Genbank: MW760398), the plasmid pET28a-APPV E2 was constructed, which contained 6 × His tag at the C terminus without endogenous transmembrane domain, and was maintained in our laboratory. The plasmid pET28a-APPV E2 was transformed into *E. coli* BL21 (Biobw, Beijing, China, bio- 82078) and identified by PCR and sequencing.

### 2.3 Detection of APPV E2 protein expression and purification of E2 protein

The *E. coli* BL21 containing plasmid pET28a-APPV E2 were harvested by centrifugation and then dissolved with the buffer (8M Urea, 50 mM Tris HCl, 300 mM NaCl, 0.1% Triton X-100, pH 8.0) and purified by Ni-NTA affinity chromatography. The purified protein was dialysis with the buffer (50mM Tris, 300mM NaCl, 0.1% SKL, pH8.0), then concentrated with PEG20000.The protein concentration was analyzed using BCA protein quantitative kits (Thermofisher). Then, protein was analyzed by Western blot. The Anti-His Tag(Abbkine, Wuhan, China, ABT2050)was used as the primary antibodies at a diluted dilution of 1:2000. And horseradish peroxidase (HRP)-conjugated goat anti-mouse IgG (Boster Biological Technology, Wuhan, China) was used as the secondary antibody at a dilution of 1:150.

### 2.4 Vaccine manufacturing

The purified APPV E2 protein was diluted to an appropriate concentration with sterile PBS, then was emulsified with ISA 201VG adjuvant (Seppic, Paris, France) at a ratio of 1:1 (w/w), IMS 1313VG adjuvant (Seppic, Paris, France) at a ratio of 1:1 (v/v), and Montanide GEL 02 adjuvant (Seppic, Paris, France) at a ratio of 95:5 (v/v), in accordance with the manufacturer’s instructions. Finally, the protein content of vaccine is 100 µg/ml.

### 2.5 Immunization of pig

All procedures were performed following the protocols approved by the animal ethics and welfare committee of Qingdao Agricultural University. The pigs, which were 3–4 weeks old and tested negative for the pathogens and antibodies of PCV2, PRRSV, PPV, HPS, and APPV, were obtained from a commercial pig farm. The pigs were divided into four groups, and five pigs were in each group. Group A, the negative control group, was intramuscular injected with 2 mL sterile PBS. Group B was intramuscularly vaccinated with 2 mL per serving (ISA 201VG adjuvant). Group C was intramuscularly vaccinated with 2 mL per serving (IMS 1313VG adjuvant). Group D was intramuscularly vaccinated with 2 mL per serving (Montanide GEL 02 adjuvant). All pigs were injected with the same vaccine dose at 21 days post-primary immunization (dpi). Serum samples were collected at 0, 7, 14, 21, 28, and 35 days post-primary vaccination, and stored at −20°C until use.

### 2.6 Antibody response

Anti-E2 detection in the serum samples obtained from vaccinated pigs, was performed using ELISA. Ninety-six-well flat-bottomed plates were coated with purified E2 protein (0.3125 μg/well), diluted in 0.05 mol/L carbonate buffer (pH 9.5), and placed at 4 °C overnight. The plate was washed three times and blocked with PBS containing 1% BSA (0.01 mol/L, pH 7.2). After washing with PBST three times, the serum samples diluted with PBST (1:200) were added to the wells and incubated at 37°C for 2 h. The plate was washed again and incubated with HRP-conjugated goat anti-pig IgG (1:8000) (Bethyl, USA) at 37 °C for 1h. TMB (3,3´,5,5´-tetramentylbenzidine) (Thermo Scientific) was added to the plate and incubated for 15 minutes at room temperature. The reaction was stopped by adding 2 M H2SO4 solution, and read at 450 nm in a µQuant micro-plate spectrophotometer (Tecan Austria GmbH). Each sample was repeated three times for the ELISA. Finally, total titers were determined by S/N (S/N=the OD450 value of samples/the OD450 value of negative control serum).

### 2.7 Challenge experiment

Whole blood, which was negative for the virus of CSFV, PCV2, PRRSV, and PPV and positive for the APPV virus, from an onset of pig from Guangzhou, was filtered (through 0.22 μm) and diluted 1:3 by sterile PBS (pH 7.2). The virus content was determined by RT-qPCR, CT = 19.17, then stored at -80°C. On the 14 days after the booster vaccination, the whole blood containing APPV was thawed and held on ice, all pigs in Group A (PBS control group) and B (ISA 201VG adjuvant vaccine group) were challenged by intramuscular with 3 mL/pig. After challenge, all pigs were monitored daily, and rectal temperatures were taken each day until 14 days. The animals were euthanized by administering pentobarbital sodium (150 mg/kg) via IV injection, blood and organs (heart, liver, spleen, lungs and kidneys, submandibular lymph nodes, inguinal lymph nodes, intestine, hip muscles and cerebellum) were collected from each pig on the 14th day.

### 2.8 Quantitative real-time polymerase chain reaction

A qRT-PCR targeting the NS3 region of the genome of APPV was used in this study (F:5’-ATTGGCTGTCTGAGGACTTCG -3’, R:5’-GAGTATCCCAAGCTTTA GTGTC-3’). One step TB Green^®^ PrimeScriptTM RT-PCR Kit II (Takara, Japan) was carried out in a 20µl reaction containing 2µl of RNA, 0.8 µl of each primer (10µM), 10 µl of 2X One step TB Green RT-PCR buffer, 0.8µl of PrimeScript 1 Step Enzyme Mix, 0.4 µl of ROX Reference Dye II, 5.2 µl of RNase Free dH2O. The reaction took place using American Applied Biosystems (ABI) 7500 real-time fluorescent quantitative PCR detection system under the following conditions: initial reverse transcription at 42°C for 5 min, followed by initial denaturation at 95°C for 10s, 40 cycles of denaturation at 95°C for 5 s and annealing and extension at 60°C for 34 s. The CT values were detected. A cut-off for positive samples was established at CT values lower than 36, according the previous research (Gatto et al., 2018).

### 2.9 Histopathologic examination

Pathological tissue samples including inguinal lymph node, submandibular lymph node, spleen, liver, kidneys and cerebellum were obtained during necropsy from each animal. All the samples were cut into approximately 1.5 cm^3^ and fixed in 4% neutral paraformaldehyde phosphate buffer (pH 7.3) about 72h. After fixation, the tissue blocks are rinsed with running water, followed by dehydration using a graded alcohol series, and then embedded to prepare paraffin sections (5µm). Subsequently, the tissue sections were stained with hematoxylin and eosin (H&E; Sigma-Aldrich, USA) and examined under a light microscope (OLYMPUS, Japan) to evaluate their pathological changes.

### 2.10 Statistical analysis

All statistical analyses were performed using IBM SPSS (SPSS Inc, Chicago, IL, USA). The APPV E2-specific antibody among the study groups are expressed as the mean ± standard deviation (SD). Statistical significance was determined by using one-way ANOVA. A p*-*value < 0.05 was considered statistically significant.

## 3 Results

### 3.1 Analysis of nucleotides and epitopes of APPV E2

**Figure 1:**
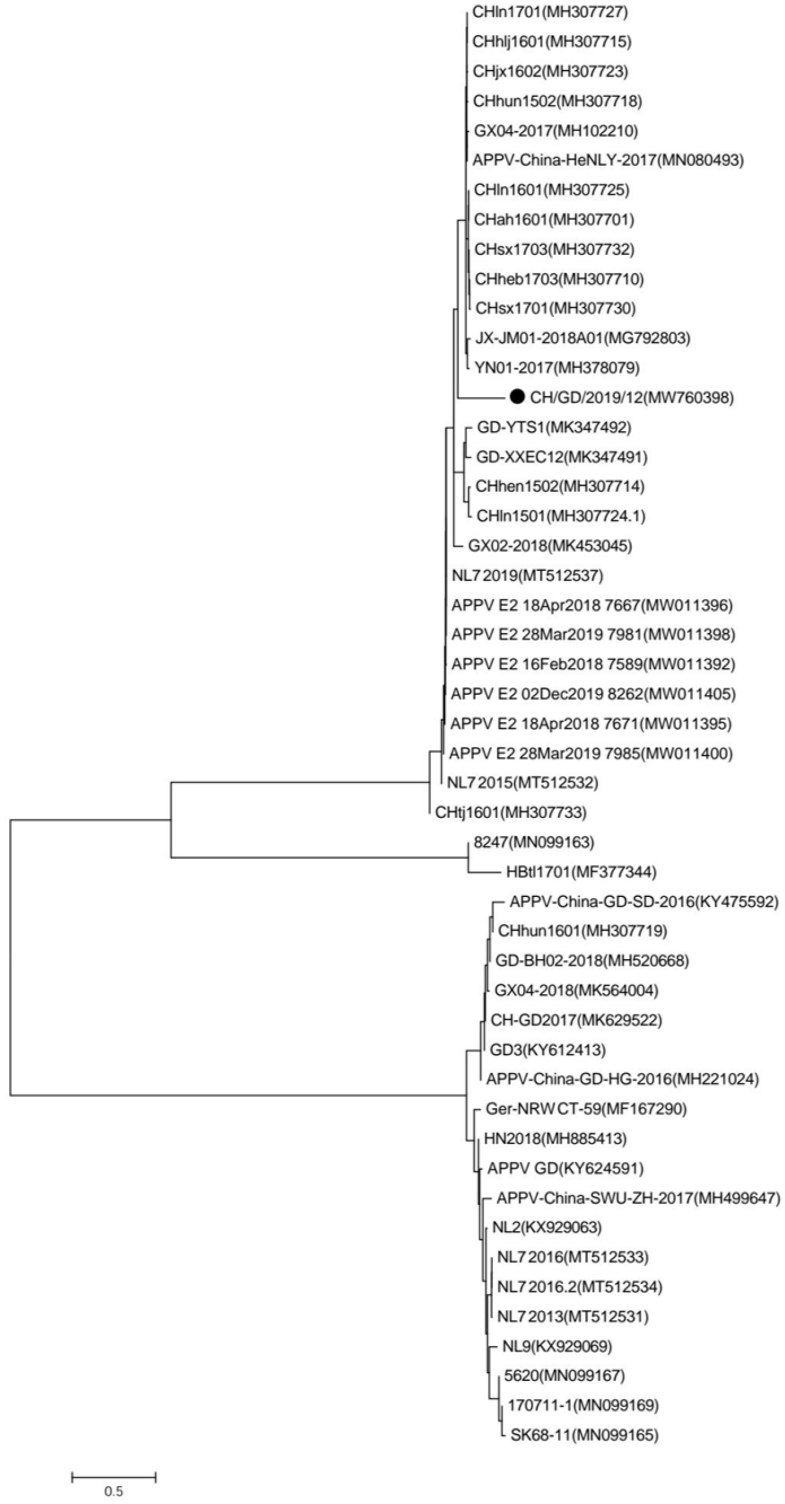
Phylogenetic reconstruction based on polyprotein sequence of E2. Indicates the CH/GD/2019/12(MW760398) described in this study.

Phylogenetic analysis was performed to determine the relationship of CH/GD/2019/12 with other APPV strains. Phylogenetic analysis based on the E2 ORF sequences indicated CH/GD/2019/12 is in a branch with other strains found in Jiangxi, Henan, Shanxi, Hebei, and other provinces in China (**Figure 1**).

### 3.2 Expression of recombinant protein

**Figure 2:**
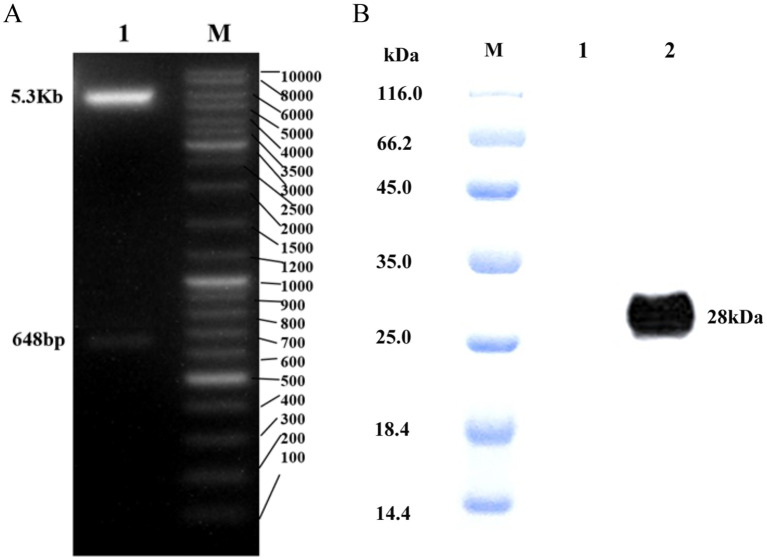
**(A)** Plasmid digestion diagram (NdeI/XhoI). M: Marker; 1: Enzyme plasmid and E2 protein. **(B)** Western blot of APPV E2 protein. M:Protein marker; 1: Negative control; 2: E2 protein.

The construction diagram of pET28a-APPV E2 is presented in **Figure 2A**. The APPV E2 protein induced by IPTG was purified using Ni-NTA chromatography and analyzed by Western blot (**Figure 2B**), showed that APPV E2 protein was successfully expressed, the size of the protein was approximately 28 kDa.

### 3.3 Specific antibodies elicited by E2 protein immunization

**Figure 3:**
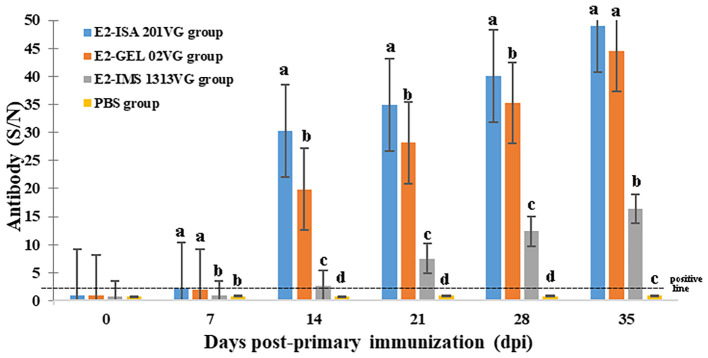
APPV E2-specific antibody detected by indirect ELISA at different dpi. Data represent the mean ± standard deviation (SD). Different letters (a, b, c) indicate a statistically significant difference between different experimental groups (*p* < 0.05). The dashed line denotes the positive threshold (S/N ratio ≥ 2.5).

As displayed in **Figure 3**, the serum titers of pigs immunized with three vaccines were significantly higher than those of the PBS control group (*p* < 0.05). The levels of APPV E2-specific antibody for Groups B (E2- IMS 201VG), and D (E2-GEL 02VG) displayed higher titers at 35 dpi (mean of S/N values were 49.00 and 44.61, respectively), the level of APPV E2-specific antibody for Group C (E2-IMS 1313VG) displayed lower titer at 35 dpi (mean of S/N value was 16.38). From 7 to 35 days after immunization, the pigs from E2- ISA 201VG group had the highest antibodies level, however, the antibody titers of pigs in control group were always negative (S/N < 2.5).

### 3.4 Temperature and clinical symptoms after APPV challenge

From 0 ~ 14 dpi, the body temperature of all the challenged pigs, including the immune group and the control group, was normal (38.7 °C ~ 39.4 °C) and had no significant difference clinical symptoms.

### 3.5 APPV virus in the organs

**Figure 4:**
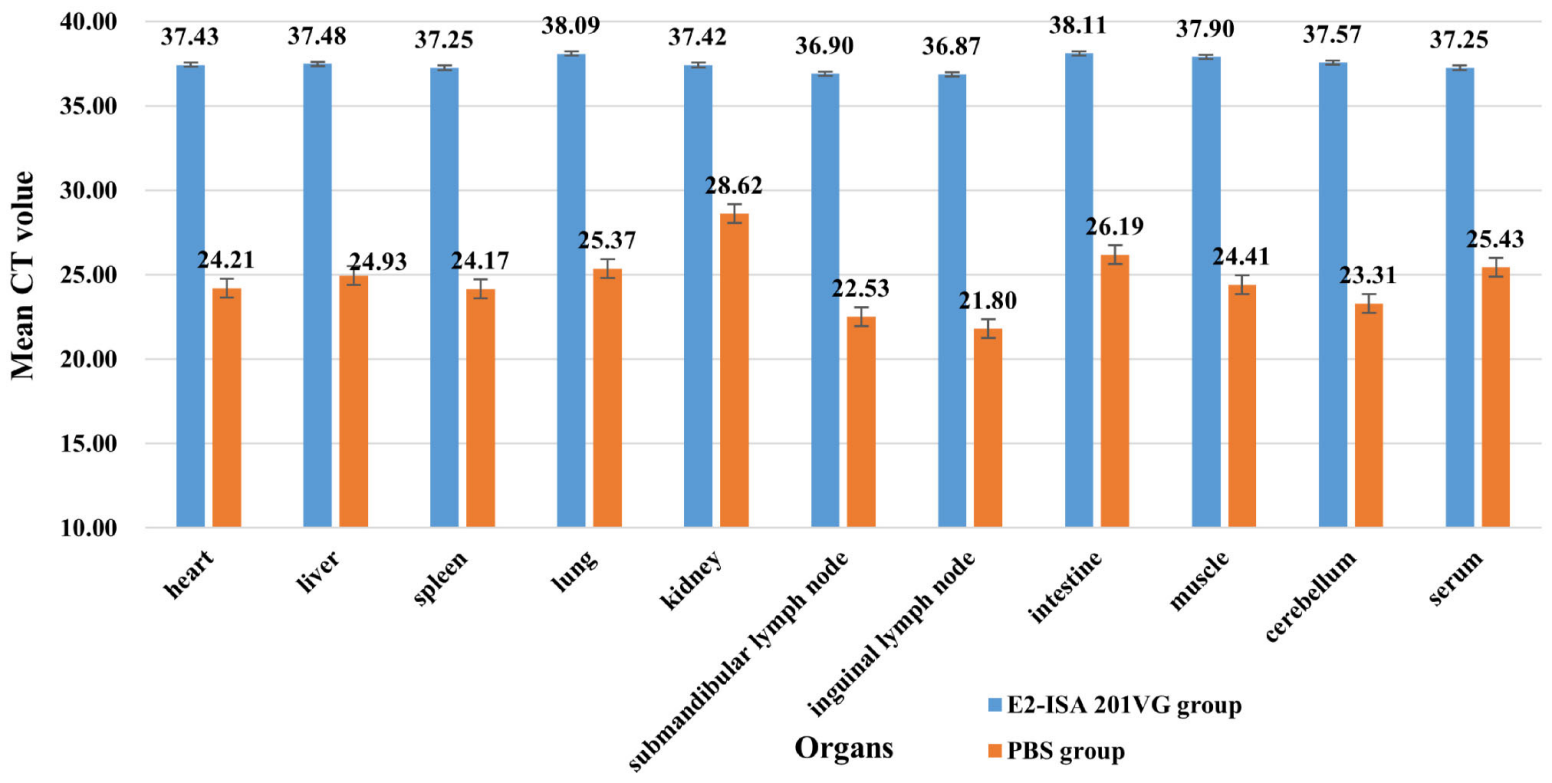
Atypical porcine pestivirus RNA detected in piglet organs after challenge by qRT-PCR.

From **Figure 4**, we see that in all organs from the PBS group, the CT values were all positive, which indicated that organs had higher viral titer (CT < 29, 21.80 ~ 28.62). In this, the APPV in lymph nodes (inguinal lymph nodes and submandibular lymph nodes) were the highest (CT = 21.80 and CT = 22.53, respectively), followed by that of the cerebellum (CT = 23.31), spleen (CT = 24.17), and heart (CT = 24.21). From the E2-ISA 201VG group results, the CT values were all negative, the APPV of all the organs had no virus (CT > 36, 36.87~38.11).

### 3.6 Histopathological changes of organs after challenge

**Figure 5:**
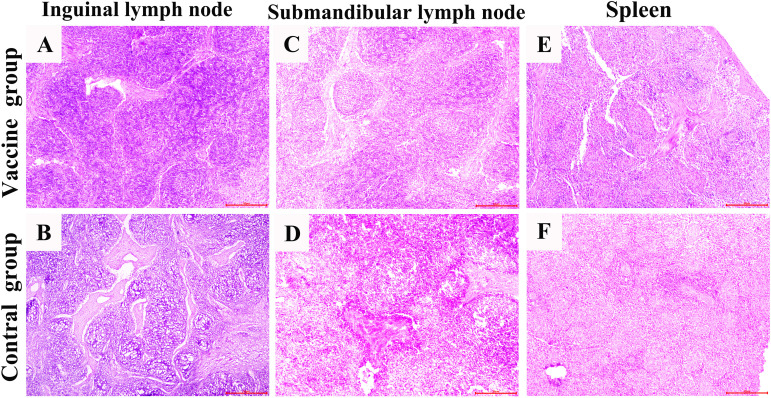
Pathological structure of secondary immune organs in the control and experimental groups. Figures **A**, **C** and **E** show that the histological structures of the samples from the vaccine group (piglets vaccinated with ISA 201VG vaccine) are all normal. Figures **B**, **D** and **F** show that the tissue structures of the samples from the control group (piglets only vaccinated with PBS) have obvious pathological changes.

**Figure 6:**
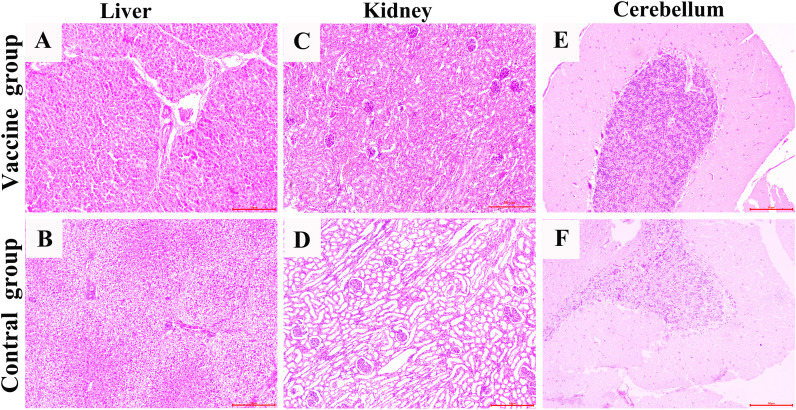
Pathological structure of liver, kidney and Cerebellum. Figures **A**, **C** and **E** show that the histological structures of the samples from the vaccine group (piglets vaccinated with ISA 201VG vaccine) are all normal. Figures **B**, **D** and **F** show that the tissue structures of the samples from the control group (piglets only vaccinated with PBS) have obvious pathological changes.

The histopathological results showed that, compared with the vaccine group, the inguinal lymph nodes, submandibular lymph nodes, spleen, liver, kidneys and cerebellum in the control group, which infected with APPV showed serious histopathological damage (**Figures 5**, **6**, **Supplementary Figures 1**-**3**). Microscopic examination revealed connective tissue hyperplasia and thickening of the trabeculae within the inguinal and submandibular lymph nodes, with destruction of the original architecture of the lymphatic follicles. Lymphoid tissue exhibits severe edema and vacuolation, where lymphocytes have disappeared and been replaced by reticular tissue, indicating chronic connective tissue hyperplasia. The spleen exhibited a marked reduction of white pulp and thinning of the periarterial lymphatic sheath, accompanied by prominent connective tissue hyperplasia in the red pulp and a significant decrease in lymphocyte density. The liver showed severe vacuolar degeneration involving most of the parenchyma and poorly defined hepatic lobules. Most of the lining epithelial cells of the renal tubules have atrophied and undergone simplification, causing dilation of the tubular lumina. Significant lesions were also observed in the control group’s cerebellum, with loosened architecture and mild edema in both cortex and medulla. The Purkinje cells lost their distinctive architecture. It should be noted that no significant inflammatory cell infiltration or extensive hemorrhage was detected throughout all samples examined.

## 4 Discussion

CT commonly occurs in newborn piglets and is characterized by tremors of the head and limbs that vary in severity. APPV has been detected in piglets with CT, and strong evidence displayed that APPV induces CT in piglets (Gatto et al., 2019). An increased number of cases of APPV has been reported in various countries all globally since 2015 (Hause et al., 2015). Reports showed that APPV has been prevalent in China since 2016 (Gatto et al., 2018; Zhang et al., 2019; Hill et al., 2024; Bergfeldt et al., 2025), we isolated APPV from a piglet in 2019, the results of nucleotide homology analysis demonstrated that this isolate has high homology with other strains from other provinces in China. APPV caused serious economic losses in pig farms (Michelitsch et al., 2019; Stenberg et al., 2020), but there are no commercialized vaccines, most of which are in the laboratory research stage. So, it is of great significance to study the vaccine against APPV in China.

Zhang et al. (2018) developed an effective subunit vaccine against APPV based on the E2 protein, which was expressed by the baculovirus expression system, which confirmed E2 protein is the main immunogenicity protein of APPV. E2 gene also serves as an important basis for genetic evolution analysis of APPV (Gatto et al., 2018). Thus, in this study, we also chose E2 as the expression protein. Regarding the choice of the expression system, the E. coli expression system has many advantages, such as safe and stable product expression, high expression volume, and easy operation. The baculovirus-insect expression system is a eukaryotic expression system that can insert many exogenous genes, but its technical operation is complex, prone to false positives, with low stability between production batches, and high difficulty in large-scale production. Thus, we chose the E. coli expression system. In this study, pET28a-APPV-E2 was successfully constructed, and western blot analysis revealed that the APPV E2 protein molecular weight reached 28 kDa, which is similar to the predicted molecular weight of APPV E2 protein (28.5 kDa) (Zhang et al., 2018).

APPV E2 protein was purified using a dialysis enrichment process. The recombinant E2 protein was immunogenic in pigs. The results of the animal experiment demonstrated that the APPV E2 protein emulsified with an adjuvant can induce high levels of specific antibodies in pigs detected by E2-ELISA. The E2-ISA 201VG and E2-GEL02VG subunit vaccine produced significantly higher antibody titers than the E2-IMS 1313VG subunit vaccine, indicating that the ISA 201VG and GEL02VG adjuvants had good immunogenicity when blinded to E2 protein. This was similar to previous research (Jang et al., 2011; Dar et al., 2013; Osman et al., 2020). These results suggested that the APPV E2 subunit vaccine induced an immune response in piglets, and ISA 201VG and GEL02VG were the dominant adjuvants. This study for the first to revealed that GEL02VG adjuvant can be tried as a preferred adjuvant for APPV vaccine.

After the challenge, the virus content of the immune group was negative, however the PBS group was opposite, indicating that E2 protein could produce a better protective effect. Previous studies demonstrated that high virus loads were identified in glandular epithelial cells, follicular centers of lymphoid organs, the inner granular cell layer of the cerebellum, and in the trigeminal and spinal ganglia (Postel et al., 2016). Similar to the previous study, we found that the APPV in lymph nodes (submandibular lymph nodes and inguinal lymph nodes) was the highest. Pathological results revealed significant structural alterations in the lymph nodes of the control group. The coexistence of high viral loads and tissue destruction strongly suggests that APPV infection may impair both local and systemic immune responses in the host. This aligns with recent findings by Hill et al. (2024), who reported that APPV infection could exacerbate the severity of Porcine Reproductive and Respiratory Syndrome (PRRS), indicating the potential immunosuppressive effects of APPV. The cerebellum is a key center for maintaining bodily balance and regulating muscle tone. Congenital tremor, the most characteristic clinical symptom of APPV infection, is pathologically based on hypomyelination in the central nervous system. Bergfeldt et al. (2025) demonstrated that the highest viral load of porcine pestivirus was detected in the cerebella of congenitally trembling piglets aged 3–4 weeks to 5 months, accompanied by marked hypomyelination and vacuolation in the cerebellar white matter. Although we did not observe very pronounced clinical symptoms in the control and vaccine groups, compared to the normal tissue structure in the cerebellum of the vaccine group, the control group exhibited significant white matter vacuolation in the cerebellum. Furthermore, a high viral load was detected in the cerebella of APPV-infected piglets in the control group, which was substantially higher than that in the vaccine group, demonstrating the direct invasiveness of APPV into the cerebellum. APPV can breach both immune and blood-brain barriers, leading to neurological symptoms in affected pigs.

In the control group of pigs, this study observed significant histopathological alterations not only in the lymph nodes and cerebellum but also notable structural lesions in organs including the spleen, liver, kidney, and heart. These findings align with the results reported by Liu et al. (2019) on the histological distribution of APPV in naturally infected piglets. However, a notable discrepancy emerged in viral load distribution: while their study identified the highest viral load in the cerebellum, our data demonstrated that the lymph nodes exhibited the highest viral load. We speculate that this divergence may be attributed to differences in infection status, such as natural versus experimental infection. However, why the virus content is highest in lymphatic organs and the pathogenesis of the disease require further research.

## 5 Conclusions

In conclusion, in our study, we constructed the recombinant E. coli APPV-E2 successfully for the first, and established E2-ELISA for the detection of antibody of vaccines. Our findings demonstrate that the APPV E2 protein can induce a robust humoral immune response and has the ability to against APPV infection in pigs, and ISA 201 and GEL02VG adjuvants can be used as candidate adjuvants for vaccine preparation. The immune principle of the APPV E2 subunit vaccine in pigs will be conducted in further studies.

## Data Availability

The original contributions presented in the study are included in the article/**Supplementary Material**. Further inquiries can be directed to the corresponding authors.
