# Supplementary material for: Immunogenicity of subunit vaccine of E2 protein against atypical porcine pestivirus in pigs
Source: Front Cell Infect Microbiol. 2026 Jan 6;15:1740259. doi: 10.3389/fcimb.2025.1740259 (PMC12816379; doi:10.3389/fcimb.2025.1740259)
Supplement: Supplementary file 1 [file Image1.pdf]

Supplementary Figures:

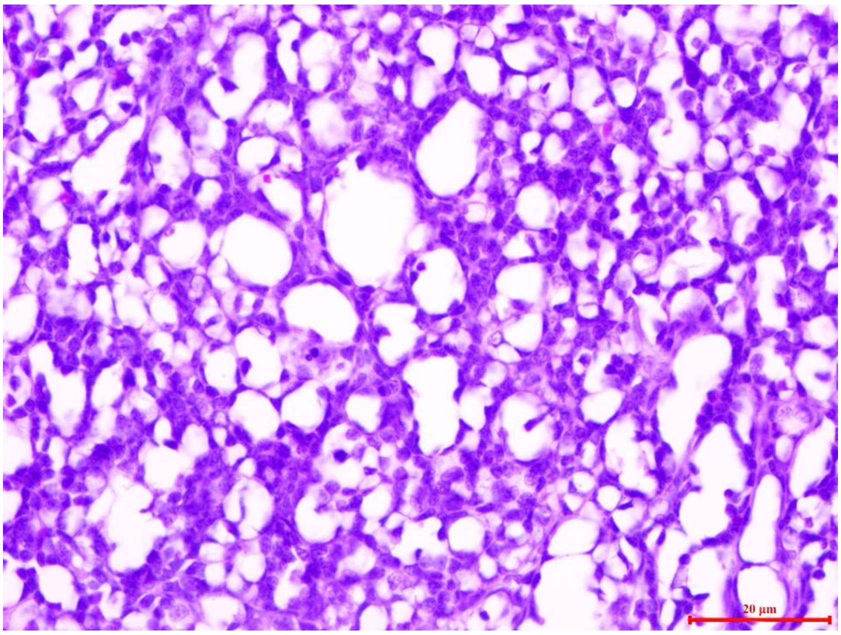

Figure 1. High-Magnification histological structure of an inguinal lymph node in control group. Lymphoid tissue exhibits severe edema and vacuolation, where lymphocytes have disappeared

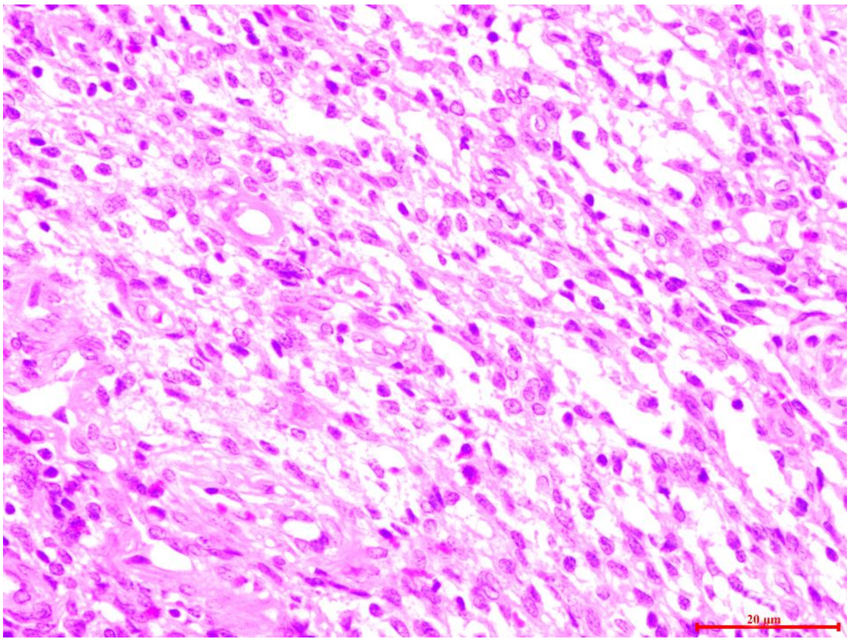

Figure 2. High-Magnification histological structure of the submandibular lymph node in control group. Lymphocytes are decreased, accompanied by an increase in reticular tissue, demonstrating marked connective tissue proliferation.

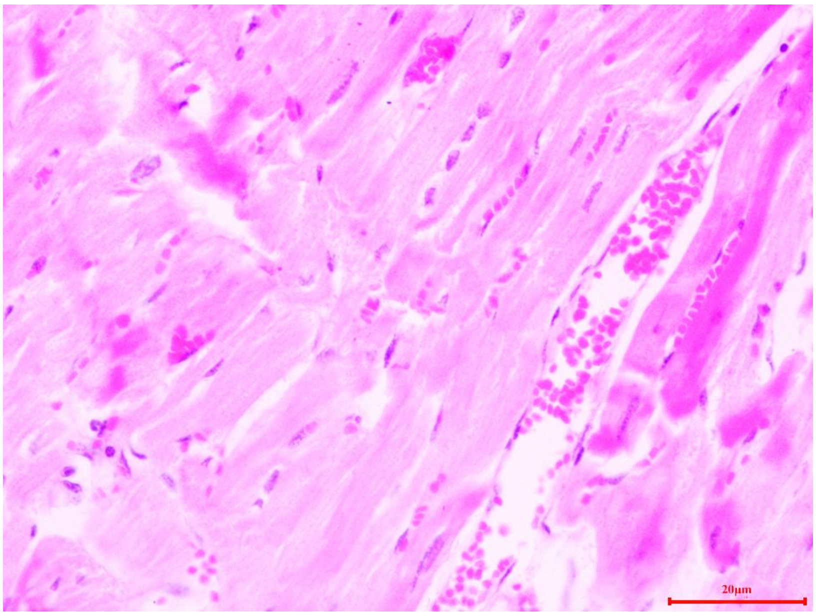

Figgure 3. High-Magnification histological structure of the myocardium in control group. The cardiac muscle cells show loss of cross-striations with indistinct intercalated discs; there is also marked hemorrhage between the myocytes.
