# Supplementary material for: Immunogenicity of subunit vaccine of E2 protein against atypical porcine pestivirus in pigs
Source: Front Cell Infect Microbiol. 2026 Jan 6;15:1740259. doi: 10.3389/fcimb.2025.1740259 (PMC12816379; doi:10.3389/fcimb.2025.1740259)
Supplement: Supplementary file 2 [file Table1.docx]

Supplementary Material

# Supplementary Data

**Table S1. 48 reference strains in this study.**

| Field strains | Orign | Genbank |
| --- | --- | --- |
| APPV_E2_18Apr2018_7671 | Netherlands | MW011395 |
| APPV_E2_18Apr2018_7667 | Netherlands | MW011396 |
| APPV_E2_28Mar2019_7981 | Netherlands | MW011398 |
| APPV_E2_02Dec2019_8262 | Netherlands | MW011405 |
| APPV_E2_28Mar2019_7985 | Netherlands | MW011400 |
| APPV_E2_16Feb2018_7589 | Netherlands | MW011392 |
| 170711-1 | Switzerland | MN099169 |
| NL2 | Netherlands | KX929063 |
| CHsx1701 | Beijing, China | MH307730 |
| NL7_2013 | Netherlands | MT512531 |
| NL7_2019 | Netherlands | MT512537 |
| NL9 | Netherlands | KX929069 |
| APPV_GD | Guangdong | KY624591 |
| 5620 | Switzerland | MN099167 |
| CHln1601 | Beijing, China | MH307725 |
| CHjx1602 | Jiangxi, China | MH307723 |
| CHln1701 | Beijing, China | MH307727 |
| CHsx1703 | Shanxi, China | MH307732 |
| NL7_2015 | Netherlands | MT512532 |
| NL7_2016 | Netherlands | MT512533 |
| NL7_2016.2 | Netherlands | MT512534 |
| HN2018 | Henan, China | MH885413 |
| 8247 | Switzerland | MN099163 |
| CHtj1601 | Beijing, China | MH307733 |
| Ger-NRW_CT-59 | German | MF167290 |
| CHheb1703 | Hebei, China | MH307710 |
| HBtl1701 | Beijing, China | MF377344 |
| CHhlj1601 | Beijing, China | MH307715 |
| CHhun1502 | Beijing, China | MH307718 |
| APPV-China-SWU-ZH-2017 | Sichuang, China | MH499647 |
| APPV-China-HeNLY-2017 | Henan, China | MN080493 |
| APPV E2 28MAR2019 7985 | Netherlands | MW011400 |
| YN01-2017 | Guangdong, China | MH378079 |
| GX02-2018 | Guangxi, China | MK453045 |
| GD-YTS1 | Guangdong, China | MK347492 |
| CHhen1502 | Beijing, China | MH307714 |
| CHln1501 | Beijing, China | MH307724.1 |
| APPV-China-GD-SD-2016 | Shandong, China | KY475592 |
| GD-XXEC12 | Guangdong, China | MK347491 |
| GX04-2018 | Guangxi, China | MK564004 |
| GX04-2017 | Guangxi, China | MH102210 |
| CHhun1601 | Hunan, China | MH307719 |
| GD-BH02-2018 | Guangdong, China | MH520668 |
| APPV-China-GD-HG-2016 | Guangdong, China | MH221024 |
| CH-GD2017 | Guangdong, China | MK629522 |
| GD3 | Guangdong, China | KY612413 |
| JX-JM01-2018A01 | Jiangxi, China | MG792803 |
| SK68-11 | Switzerland | MN099165 |
